# Tyrosine carbon dots inhibit fibrillation and toxicity of the human islet amyloid polypeptide†

**DOI:** 10.1039/d0na00870b

**Published:** 2020-11-10

**Authors:** Daniel Nir Bloch, Shani Ben Zichri, Sofiya Kolusheva, Raz Jelinek

**Affiliations:** Department of Chemistry, Ben Gurion University of the Negev Beer Sheva 84105 Israel razj@bgu.ac.il; Ilse Katz Institute for Nano-Science and Technology (IKI), Ben Gurion University of the Negev Beer Sheva 84105 Israel

## Abstract

Misfolding and aggregation of the human islet amyloid polypeptide (hIAPP) are believed to play key roles in the pathophysiology of type-II diabetes. Here, we demonstrate that carbon dots (C-dots) prepared from the amino acid tyrosine inhibit fibrillation of hIAPP, reduce hIAPP-induced cell toxicity and block membrane disruption by the peptide. The pronounced inhibitory effect is traced to the display of ubiquitous aromatic residues upon the C-dots' surface, mimicking the anti-fibril and anti-toxic activity of natural polyphenolic compounds. Notably, spectroscopy and thermodynamics analysis demonstrated different hIAPP interactions and fibril inhibition effects induced by tyrosine-C-dots displaying phenolic residues and C-dots prepared from phenylalanine which exhibited phenyl units on their surface, underscoring the significance of hydrogen bonding mediated by the phenolic hydroxide moieties for the fibril modulation activity. The presented experiments attest to the potential of tyrosine-C-dots as a therapeutic vehicle for protein misfolding diseases, interfering in both π–π interactions as well as hydrogen bonding involving aromatic residues of amyloidogenic peptides.

## Abstract 2

Carbon dots prepared from tyrosine and citric acid inhibit fibrillation, membrane interactions and toxicity of hIAPP.

## Graphical abstract

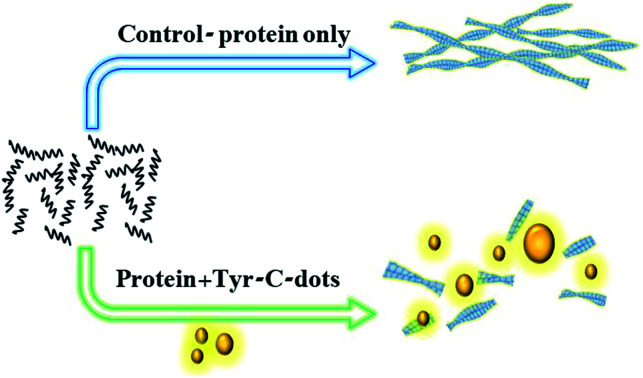

## Introduction

Protein misfolding and aggregation are the hallmarks of varied diseases and pathologies, including Alzheimer's disease (AD), Parkinson's disease (PD), and type 2 diabetes (T2D).^1^ The human islet amyloid polypeptide (hIAPP, or amylin), a 37-residue peptide secreted in the pancreatic β cells, has been identified as the main component of plaques found in the pancreas of diabetic patients.^2^ hIAPP is intrinsically disordered,^3^ undergoing a conformational change in which it adopts β-sheet structure between residues 8–19 and 25–37, further leading to fibril formation.^4,5^ hIAPP oligomers, however, are believed to be the main toxic species, likely through disruption of cell membranes.^6–8^

In light of the presumed closed relationship between hIAPP aggregation and its pathogenicity, inhibition of hIAPP self-assembly and fibril formation have been a major strategy aimed at identifying therapeutic treatments to T2D.^9^ Synthetic aggregation inhibitors such as short peptides^9,10^ metal nanoparticles^11–13^ have been reported. Polyphenols, in particular, constitute a prominent family of aggregation inhibitors.^14,15^ Curcumin and epigallocatechin gallate (EGCG), for example, inhibit aggregation and toxicity of hIAPP.^16,17^ Resveratrol has been hypothesized to reduce toxicity of hIAPP by promoting non-toxic aggregation pathways.^18,19^ In general, polyphenols are believed to modulate peptide self-assembly processes through interference in π–π interactions which play core roles in amyloid fibril formation.^20–22^ Polyphenols, however, are limited as viable therapeutic agents since they are targeted by the immune system and rapidly cleared.

Carbon dots (C-dots) are unique carbonaceous nanoparticles (<10 nm) which have attracted considerable interest due to their tunable fluorescence emission properties,^23^ low toxicity and biocompatibility.^24^ C-dots have been employed in diverse applications, including bioimaging,^25–27^ chemical and biological sensing,^28^ catalysis,^29^ and others. Recent studies demonstrated intriguing therapeutic applications of C-dots. In the context of amyloid diseases, several reports have shown that C-dots can selectively inhibit peptide aggregation.^30,31^ In particular, C-dots were shown to promote off-pathway fibrillation of hIAPP thereby blocking its toxicity through putative elimination of oligomeric peptide species.^32^ Importantly, recent studies have demonstrated that because of the mild synthesis conditions, particularly low reaction temperatures, C-dots retain “structural memory” of the carbonaceous precursors.^27,33,34^ As such, C-dots may display on their surfaces abundant functional residues imported from their molecular building blocks.^33,35^

Here, we exploited the “C-dot structural memory” concept through synthesis of C-dots from tyrosine, which display the aromatic units on their surface, effectively mimicking the aggregation inhibition properties of natural polyphenols. Indeed, the tyrosine C-dots' biocompatibility, high chemical stability and surface display of ubiquitous bioactive units is expected to contribute to the aggregation inhibitory effect. Interestingly, the experiments indicate a significant difference in hIAPP fibril modulation activity between C-dots prepared from tyrosine and C-dots synthesized from phenylalanine. Mechanistic analysis reveals that the phenol residues play significant roles in blocking hIAPP assembly, aided by hydrogen bonding of the hydroxide units in the phenol residues. Overall, this work highlights the use of C-dots as potential therapeutic platform in type-II diabetes and other protein misfolding diseases.

## Materials and methods

### Materials

human amylin (hIAPP) was purchased from AnaSpec and Lifetein in a lyophilized form at >95% purity. l-Tyrosine (Tyr) 99%, l-phenylalanine (Phe) 99% and citric acid were purchased from Alfa Aesar. Glycine 99%, Thioflavin T (ThT), sodium carbonate, sodium bicarbonate and Tris base buffer were purchased from Sigma Aldrich. Hexafluoro-2-propanol (HFIP) was purchased from Apollo scientific and HCl 32% was purchased from bio lab ltd, 1,6-diphenylhexatriene (DPH) was obtained from Molecular Probes, Inc. (Eugene, OR).

### Carbon-dot synthesis

C-dots were prepared by a one-pot synthesis method in which 1.0 g of the desired amino acid was mixed with citric acid in molar ratio of 3 : 1 and dissolved in 10 mL deionized water (18.2 MΩ cm, Thermo Scientific) while few drops of concentrated HCl were added to dissolve the precursors and obtain a clear solution. The solution was then transferred to a poly(tetrafluoroethylene) (Teflon)-lined autoclave, and hydrothermal heating was carried out at 215 °C for 17 h. Thereafter, the C-dot solutions were purified by dialysis in deionized water, subsequently subjected to lyophilization. The dry powder was re-dissolved in phosphate buffer (PB) or in Tris buffer at a concentration of 5 mg mL^−1^.

### Peptide sample preparation

hIAPP was dissolved in HFIP at a concentration of 0.5 mg mL^−1^ and stored at −20 °C until use to prevent aggregation. For each experiment, the solution was thawed and the required amount was dried by evaporation for 5–6 h to remove the HFIP. The dried peptide sample was dissolved in 10 mM sodium phosphate buffer (PB) pH 7.4, or in Tris buffer 0.05 M pH 7.4 at room temperature.

### Vesicle preparation

Vesicles comprising of DOPC were prepared by dissolving the lipids in chloroform/ethanol (1 : 1, v/v) and drying *in vacuo*. Small unilamellar vesicles (SUVs) were prepared in 10 mM sodium phosphate (pH 7.4) by probe sonication (power: 130 W, frequency: 20 kHz, at 20% amplitude) for 10 min. Vesicle suspensions were allowed to stand for 1 h at room temperature prior to usage.

### Atomic force microscopy (AFM)

The C-dot aqueous solution (80 μL, 0.05 mg mL^−1^) was deposited on a silicon wafer and scanned by AFM under wet conditions (Cypher ES, Asylum Research, an Oxford Instruments company, Goleta, CA), in tapping mode. All images were acquired using a silicon probe (AC 40, Olympus) under the following conditions: spring constant 2 N m^−1^, frequency 25 kHz, and a tip with radius 9 nm.

### Ultraviolet visible (UV-vis) spectroscopy

UV-vis spectra were acquired on a Thermo Scientific Evolution 220 spectrophotometer. All absorbance measurements were performed using 1 cm cells in water as dispersive medium.

### Fourier transform-infrared (FT-IR)

FTIR spectra were recorded on a Nicolet FTIR spectrometer (6700 FTIR spectrometer), using the attenuated total reflectance (ATR) technique with a diamond crystal, collecting data with clean crystal as a background. For each sample, a reference spectrum was first acquired from a clean crystal then the Spectra of dry samples were recorded by putting few mgs of dried sample. Analysis was carried out using Omnic (Nicolet, Madison, WI, USA) software.

### Thioflavin-T (ThT) fluorescence assay

ThT fluorescence measurements were taken at 25 °C using 96-well black plate on a Biotek Synergy H1 plate reader. Measurements were made on a sample containing 15 μM IAPP in the absence or presence of the tested C-dots. A 150 μL aliquot of the aggregation reaction was mixed with 10 μM ThT in 10 mM phosphate buffer or in Tris buffer 0.05 M (pH 7.4). The fluorescence intensity was measured every 4 min for 20 h at *λ*_ex_ = 440 and *λ*_em_ = 485 nm. The self-fluorescence of the C-dots was subtracted from measured fluorescence for each sample. Results are presented as means ± SD for each sample.

### Transmission electron microscopy (TEM)

5 μL aliquots of samples used in the ThT experiments (after 20 h incubation) were placed on 400-mesh copper grids coated with a carbon-stabilized formvar film. Excess solutions were removed following 2 min incubation, and the grids were negatively stained for 30 s with a 1% uranyl acetate solution. Samples were viewed in a FEI Tecnai 12 TWIN TEM operating at 120 kV.

### Circular dichroism (CD) spectroscopy

CD spectra were recorded in the range of 190–260 nm at room temperature on a Jasco J-715 spectropolarimeter, using 1 mm quartz cuvettes. Solutions in volumes of 400 μL contained 25 μM IAPP in the absence or presence of different C-dots in concentration of 0.05 mg mL^−1^. Spectra were recorded at times 0 h, 6 h and 24 h incubation. CD signals resulting from buffer and C-dots were subtracted from the corresponding spectra.

### Cell viability assay

SH-SY5Y neuroblastoma cell lines were a generous gift from Prof. Niv Papo (BGU). SH-SY5Y cells were grown at 37 °C and 5% CO_2_ in Dulbecco's modified Eagle medium (DMEM) supplemented with 10% tetracycline-free fetal bovine serum (FBS), l-glutamine (2 mM), and penicillin (100 units per mL)/streptomycin (0.1 mg mL^−1^) (Gibco, Israel). The effect of hIAPP and C-dots (separately and mixed together) on SH-SY5Y cells were assayed by using an XTT-based kit (biological industries). Briefly, cells were seeded (1 × 10^4^ cells per well) in a 96-well plate coated for tissue culture and incubated at 37 °C in 5% CO_2_ for 24 h. The medium was then replaced with fresh DMEM medium. The cells were mixed with Tyr C-dots in different concentrations in the presence or absence of freshly-dissolved hIAPP (final concentration was 35 μM) and further incubated for 48 h at 37 °C/5% CO_2_. Control measurements of cells in the presence of hIAPP and in buffer where also performed. 50 μL of XTT reagent were added to each well and cell viability was assessed at 490 nm and 680 nm wavelengths with 5 repeats on BioTek Synergy 4 microplate reader (Winooski, VT, USA). Data for each sample were normalized according to the cell-only control.

### Isothermal titration calorimetry (ITC)

hIAPP was dried as described above and dissolved in Tris-buffer 0.05 M, pH 7.4 or in PB buffer 0.01 M pH 7.4, the tested. C-dots were diluted with corresponding buffer to final concentration of 5.0 mg mL^−1^. Samples of 170 μL of the peptide 45 μM solution or 170 μL of the buffer were inserted into the Nano ITC low volume cell (TA Instruments, Newcastle, DE). The titrating syringe was filled with 50 μL of the tested C-dots. After equilibrium had been reached, injection of 2.5 μL aliquots was carried out every 350 s.

### Fluorescence anisotropy

The fluorescence probe DPH was incorporated into the SUVs (DOPC) by adding the dye dissolved in THF (1 mM) to vesicles up to a final concentration of 1.25 μM. After 30 min of incubation at 30 °C of DPH, fluorescence anisotropy was measured at *λ*_ex_ = 360 nm and *λ*_em_ = 430 nm using a FL920 spectrofluorimeter (Edinburgh Co., Edinburgh, UK). Data were collected before and after the addition of freshly prepared mixtures of the tested C-dot/hIAPP and incubation of 24 h. As control measurements we measured the effect of the hIAPP and C-dots alone. Anisotropy values were automatically calculated by the spectrofluorimeter software using the equation: *r* = (*I*_VV_ − *GI*_VH_)/(*I*_VV_ + 2*GI*_VH_), *G* = *I*_HV_/*I*_HH_, in which *I*_VV_ is with excitation and emission polarizers mounted vertically; *I*_HH_ corresponds to the excitation and emission polarizers mounted horizontally; *I*_HV_ is the excitation polarizer horizontal and the emission polarizer vertical; *I*_VH_ requires the excitation polarizer vertical and emission polarizer horizontal. Results are presented as means ± standard error of the mean (SEM) of seven replicates.

## Results and discussion

**Scheme 1:**
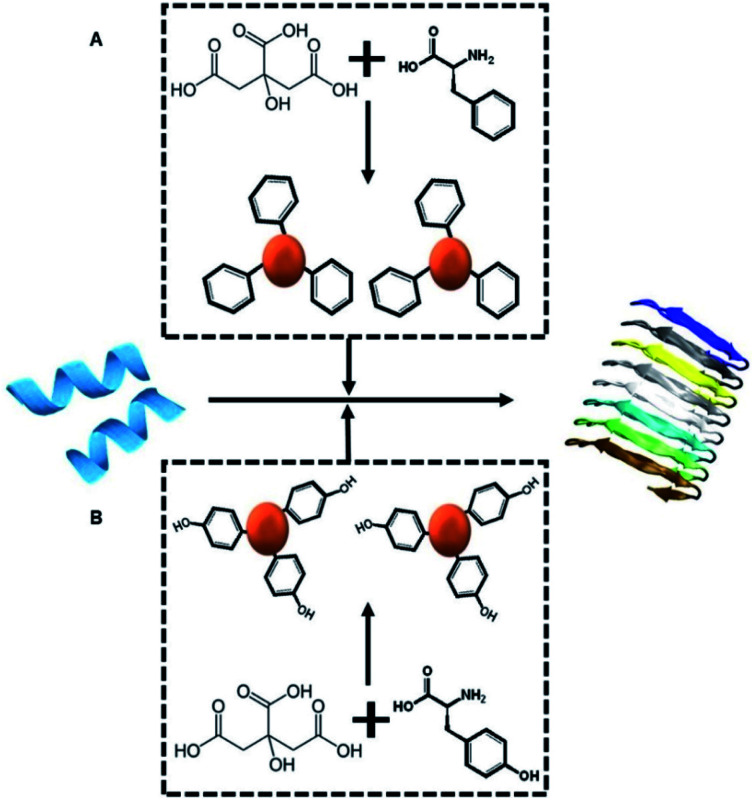
Experimental concept. Monomeric hIAPP (blue peptide, left) undergoes spontaneous assembly in buffer, forming amyloid fibrils (shown schematically on the right). We examine how co-incubating hIAPP with C-dots (red spheres) prepared from either citric acid and phenylalanine as the carbon precursors (top-A) or citric acid and tyrosine (bottom-B) might affect the fibrillation process.

Scheme 1A depicts the experimental concept. Our objective is to determine whether C-dots prepared from aromatic amino acids, specifically tyrosine (Tyr-C-dots) and phenylalanine (Phe-C-dots), affect the fibrillation properties of hIAPP and the biological implications of C-dot-modulated hIAPP aggregation. Specifically, the experiments are designed to determine whether, and to what extent, the aromatic residues displayed upon the C-dots' surfaces would interfere with hIAPP self-assembly.

The two C-dot species were prepared by a simple hydrothermal process using the amino acids as reaction precursors (see Experimental section). To assess the effects of the C-dots upon hIAPP fibrillation and biomolecular properties, they were dissolved in buffer and co-incubated with the peptide.

**Fig. 1:**
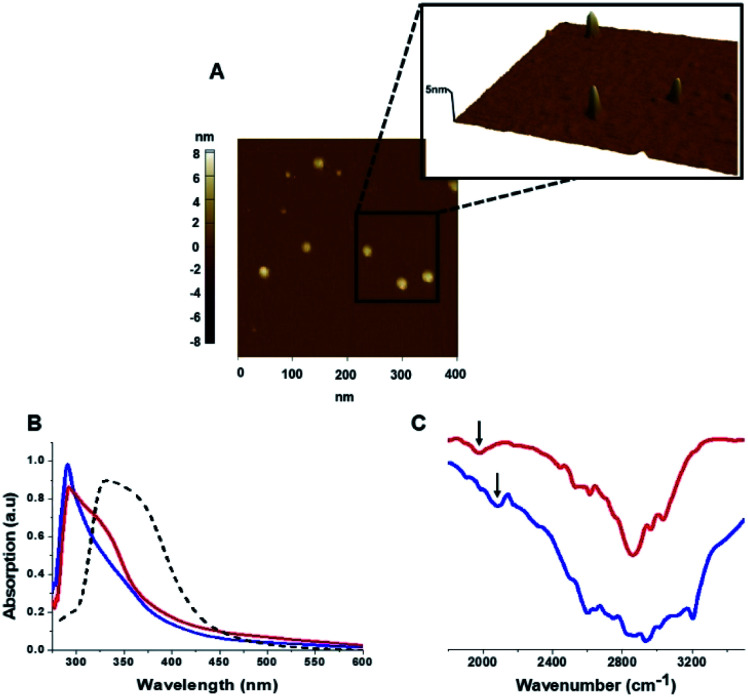
Carbon dots characterization. (A) Representative AFM image showing Tyr-C-dots; (B) UV-vis absorbance spectra of Tyr-C-dots (blue), Phe-C-dots (red), and C-dots prepared from glycine (a non-aromatic amino acid) and citric acid (broken spectrum). Absorbance peaks at around 280 nm for both Tyr-C-dots and Phe-C-dots account for aromatic residues displayed on the C-dots' surface. (C) FTIR spectra of Tyr-C-dots (blue) and Phe-C-dots (red). The arrows indicate signals ascribed to the C-dots' surface-displayed phenol (in the case of Tyr-C-dots) and phenyl (Phe-C-dots) residues.

**Fig. 2:**
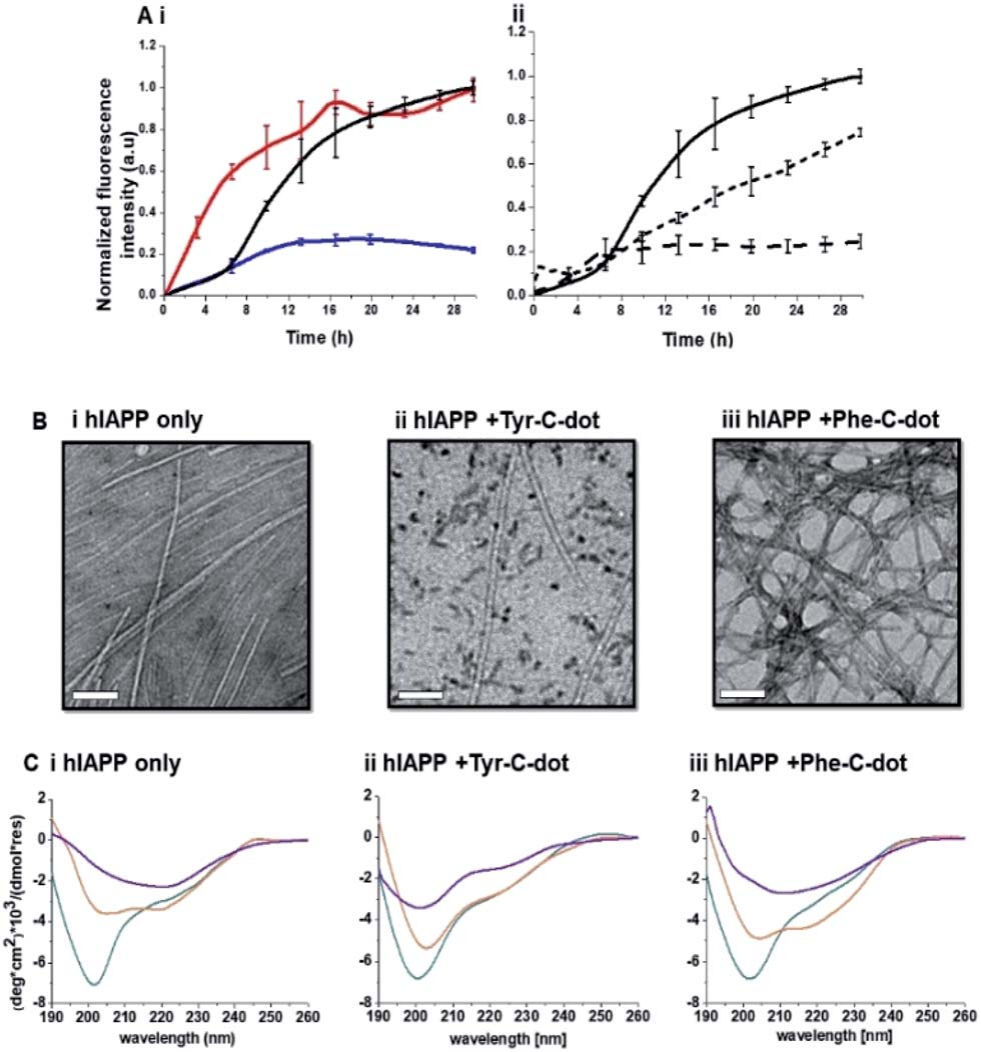
Effects of the carbon dots upon hIAPP fibrillation. (A) ThT fluorescence curves. (i) hIAPP alone (black curve); hIAPP + Tyr-C-dots (blue); hIAPP + Phe-C-dots (red). (ii) hIAPP alone (solid curve), hIAPP + Tyr-C-dots (0.01 mg mL^−1^; short dash), hIAPP + Phe-C-dots (0.03 mg mL^−1^; long dash). (B) Transmission electron microscopy (TEM) images. Bars correspond to 100 nm. (C) Circular dichroism (CD) spectra. In all panels: turquoise spectra were recorded immediately after peptide and C-dots dissolution in PB buffer; orange spectra: 6 hours after dissolution; purple spectra: 24 hours after dissolution.

**Fig. 3:**
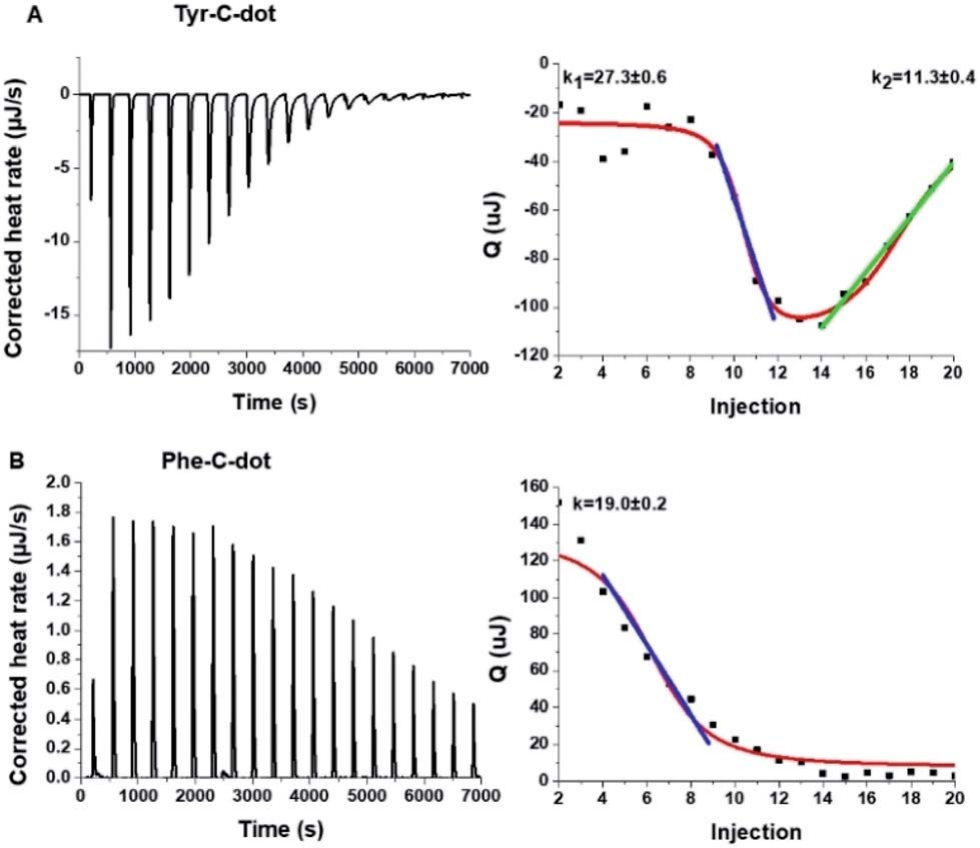
Interactions between hIAPP and the carbon dots analysed by isothermal titration calorimetry (ITC). (A) Tyr-C-dot/hIAPP; (B) Phe-C-dot/hIAPP. Left: Titration data after subtraction of the buffer. Right: Heat capacity *vs.* injection numbers.

The C-dots were characterized by several analytical techniques (Fig. 1). Fig. 1A presents a representative atomic force microscopy (AFM) image of the Tyr-C-dots, indicating relative homogeneity of the C-dots (similar AFM data were obtained for the Phe-C-dots). Size distributions of the C-dots, based upon statistical analyses of the AFM data, were 5 ± 2 nm for the Tyr-C-dots and 6 ± 2 nm in the case of Phe-C-dots (Fig. 1, SI†). The fluorescence spectra in Fig. 2, SI† feature the typical excitation-dependent emission profiles of the C-dots, while X-ray photoelectron spectroscopy (XPS) analysis (Fig. 3, SI†) further accounts to the atomic units associated with C-dot formation.

The ultraviolet-visible (UV-vis) absorbance spectra in Fig. 1B furnish evidence for the presence of the aromatic residues originating from tyrosine and phenylalanine on the C-dots' surfaces. Specifically, the UV-vis spectra of both Tyr-C-dots and Phe-C-dots feature a prominent peak at around 280 nm ascribed to the phenol or phenyl units.^36^ For comparison, we additionally prepared C-dots using glycine, a non-aromatic amino acid, and citric acid, a non-aromatic amino acid, as the carbonaceous precursor. Indeed, the UV-vis spectrum of the Gly-C-dots did not exhibit absorbance in the aromatic region around 290 nm (dashed spectrum in Fig. 1B). Rather, the peak at around 350 nm for the Gly-C-dots is attributed to the graphitic cores of the C-dots; the graphitic units are similarly reflected in the shoulder and broad slope at around 350 nm recorded in the case of Tyr-C-dots and Phe-C-dots, respectively (Fig. 1B).

Fig. 1C presents the Fourier transform infrared (FTIR) spectra of the Tyr-C-dots and Phe-C-dots further illuminating the molecular species displayed upon the C-dots' surface. The FTIR spectrum of Tyr-C-dots (Fig. 1C, blue) features a broad peak between 2400–3300 cm^−1^ corresponding to hydroxyl moieties, while the signal at ∼2000 cm^−1^ is ascribed to the phenol residues.^37,38^ The FTIR spectrum of the Phe-C-dots (Fig. 1C, red) reveals an aromatic peak shifted to a lower frequency (indicated by the arrow), corresponding to the phenyl residues displayed on the surface of the Phe-C-dots. Indeed, the overall higher intensities of the FTIR spectral features between 2500–3300 cm^−1^ for Tyr-C-dots account for the ubiquitous OH units upon the C-dots' surface.

Fig. 2 depicts experiments designed to examine the impact of Tyr-C-dots and Phe-C-dots upon self-assembly and fibril formation by hIAPP. The thioflavin-T (ThT) fluorescence curves in Fig. 2A and B reveal significant difference between the effects of Tyr-C-dots and Phe-C-dots upon hIAPP fibrillation. ThT fluorescence is widely used to monitor fibril formation as the ThT dye intercalates within β-sheet units of peptide fibrillar structures, giving rise to enhanced fluorescence emission.^39,40^ The ThT fluorescence curve of hIAPP alone (Fig. 2A,i, black curve) features a well-known initial lag time and steep increase before reaching a plateau.^13,41,42^ In comparison, the ThT fluorescence curve recorded in case of the hIAPP/Tyr-C-dot mixture (Fig. 2A,i, blue curve) demonstrates that Tyr-C-dots co-incubated with hIAPP significantly inhibited fibril assembly. Phe-C-dots, however, had seemingly the opposite effect, reducing the lag time prior to fibril formation while reaching the same ThT fluorescence intensity plateau as the control hIAPP sample (Fig. 2A,i, red curve). The fibrillation inhibition effect of Tyr-C-dots is also manifested in the ThT fluorescence analysis in Fig. 2A,ii showing inverse relationship between ThT fluorescence emission and Tyr-C-dot concentration.

Transmission electron microscopy (TEM) analysis in Fig. 2B illuminates the divergent effects of the two types of C-dots upon hIAPP fibrillation. Specifically, when Tyr-C-dots were co-incubated with hIAPP, significantly fewer fibrils were observed in the TEM analysis (Fig. 2B,ii) compared to the hIAPP control sample (Fig. 2B,i). This result is consistent with the ThT data in Fig. 2A reflecting inhibition of fibril formation by the Tyr-C-dots. Notably, ubiquitous small aggregates were also apparent in the TEM image of the Tyr-C-dot/hIAPP mixture (Fig. 2B,ii), likely accounting for inhibited assembly of mature fibrils. In comparison, when hIAPP was co-incubated with Phe-C-dots, a dense fibril network was apparent (Fig. 2B,iii). This result similarly echoes the ThT fluorescence curve in Fig. 2A,i, pointing to slight acceleration of hIAPP upon co-incubating with Phe-C-dots.

**Fig. 4:**
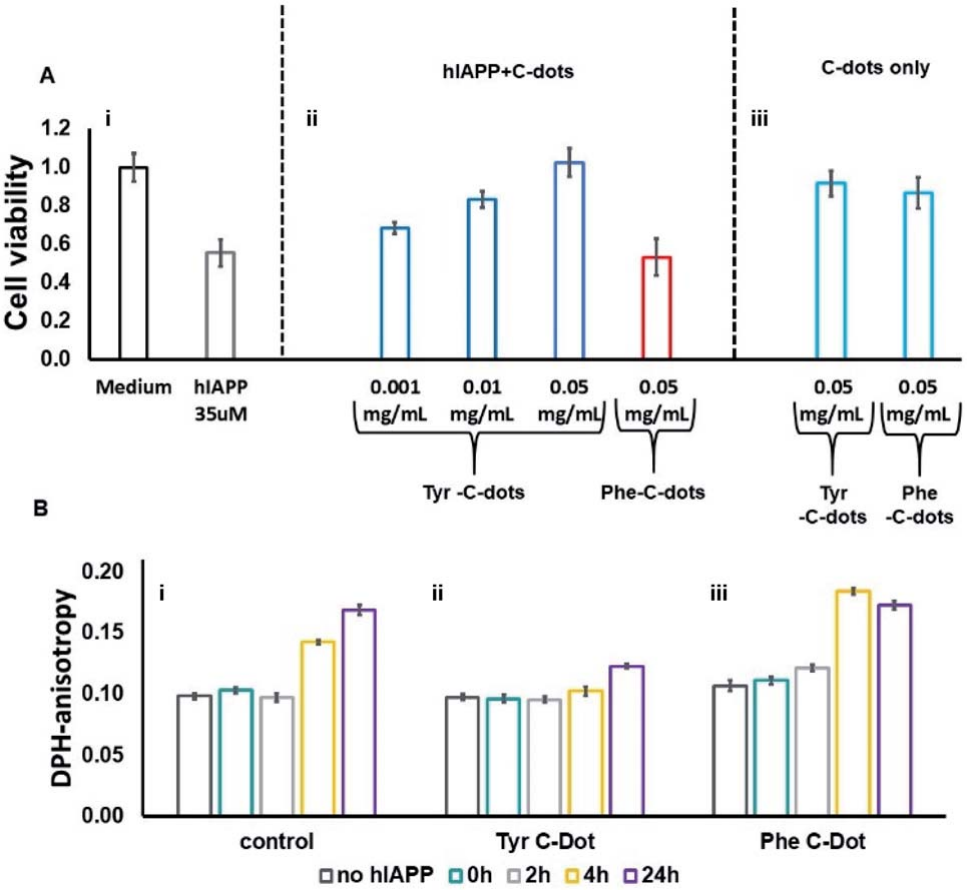
Effects of the carbon dots on hIAPP-induced cell toxicity and membrane interactions. (A) SH-SY5Y cell viability determined by the XTT assay, hIAPP concentration was 35μM. (B) DPH fluorescence anisotropy in DPH/DOPC (1 : 500 mole ratio) vesicles. C-dot final concentration used in all measurements was 0.03 mg mL^−1^ and the hIAPP concentration was 15 μM.

The circular dichroism (CD) spectroscopy experiments in Fig. 2C complement the ThT and TEM results, attesting to distinct modulations of hIAPP self-assembly by the C-dots. The CD spectra of the control hIAPP sample in Fig. 2C,i (without addition of C-dots) indicate that the peptide had an initial random coil conformation (Fig. 2C,i turquoise spectrum), gradually adopting a β-sheet conformation, which was the predominant structure after 24 hours (reflected in the minimum at ∼230 nm; purple spectrum, Fig. 2C,i).^42,43^ When Tyr-C-dots were co-incubated with hIAPP, however, structural transformation of the peptide did not occur (Fig. 2C,ii) and the random coil conformation was retained even after 24 hours (Fig. 2C,ii purple spectrum). This result is consistent with inhibition of the fibril assembly process by the C-dots, as apparent in the ThT (Fig. 2A) and TEM (Fig. 2B) experiments. In the case of Phe-C-dots, the time-dependent CD signatures of hIAPP were similar to the control experiment (Fig. 2C,iii), echoing the ThT and TEM data recorded for the Phe-C-dot/hIAPP samples (Fig. 2A and B) indicating no inhibition of fibril formation by the Phe-C-dots. Importantly, Tyr-C-dots also induced disintegration of mature hIAPP fibrils (Fig. 4, SI†).

To shed light on the interactions between the C-dots and hIAPP, isothermal titration calorimetry (ITC) measurements were carried out (Fig. 3). The ITC experiments were aimed at illuminating the distinct effects of the Tyr-C-dots and Phe-C-dots, respectively, upon fibrillation of the peptide. In the experiments, the C-dots were titrated into a hIAPP solution and the integrated injection heat values were obtained through subtracting the buffer titration points from peptide titrations for the two C-dot systems. The graphs depicting heat capacity *vs.* injection numbers in Fig. 3 (right) indicate a significant difference in the thermodynamic profiles and binding modes between the two C-dot/hIAPP systems. Specifically, the V-shape appearance of the Tyr-C-dot/hIAPP ITC curve (Fig. 3A, right) accounts for two distinct binding modalities, an initial endothermic interaction and a subsequent exothermic binding process. The two slopes (*k*_1_ and *k*_2_ indicated in Fig. 3A) calculated for the two processes are outlined in Fig. 3.

The ITC titration curve recorded in the case of Phe-C-dot/hIAPP (Fig. 3B) attests to significantly different interactions between the Phe-C-dots and the peptide in comparison with Tyr-C-dots. Specifically, the ITC curve features an endothermic sigmoidal shape accounting for a single binding process between the Phe-C-dots and hIAPP, different than the two-step processes recorded in the case of Tyr-C-dots (*e.g.*Fig. 3A). The significant difference between the hIAPP binding modes of the two C-dot species echoes their divergent fibrillation effects (*e.g.*Fig. 2), underscoring the critical role of the phenol moieties at the Tyr-C-dots' surface in C-dot/peptide interactions.

hIAPP fibrillation, particularly membrane disruption by oligomeric species, has been linked to pathophysiological effects and cellular toxicity. Accordingly, Fig. 4 presents the effect of the C-dots upon hIAPP-induced cell toxicity and bilayer interactions. The bar diagram in Fig. 4A depicts the effects of hIAPP and the C-dots upon SH-SY5Y cell viability, determined by the XTT assay. hIAPP alone (at a concentration of 35 μM) reduced cell viability by almost 50%, reflecting its well-known cytotoxicity.^44,45^ Co-addition of Tyr-C-dots and hIAPP to the cells, however, exhibited significantly lower cell toxicity (Fig. 4A). Indeed, cell protection was directly dependent upon the concentration of the Tyr-C-dots; viability that was similar to the control cells was retained at a Tyr-C-dot concentration of 0.05 mg mL^−1^. In contrast, Fig. 4A shows that the Phe-C-dots did not affect hIAPP-induced cell toxicity, even at a high concentration (0.05 mg mL^−1^) in which the Tyr-C-dots blocked the toxic effect of the peptide. The dramatically divergent effects of the two C-dots upon hIAPP-induced toxicity are consistent with the pronounced difference between their effects upon hIAPP fibrillation (Fig. 2), and the distinct C-dot–peptide interactions (Fig. 3).

The toxic effects of hIAPP have been linked to perturbation of the cellular membrane by the peptide, specifically involving its oligomeric aggregates.^46,47^ As such, we also assessed the impact of the C-dots upon the interactions of hIAPP with lipid bilayers (Fig. 4B). Specifically, in the experiments depicted in Fig. 4B we prepared vesicles comprising DOPC and the fluorescence dye diphenylhexatriene (DPH), and measured the fluorescence anisotropy of DPH, a known marker of bilayer dynamics.^48^ In particular, DPH anisotropy modulation illuminates the effect of membrane-active compounds upon bilayer structure and dynamics.^48–50^ The bar diagram in Fig. 4B,i demonstrates that when hIAPP alone was incubated with the DPH/DOPC vesicles the DPH fluorescence anisotropy significantly increased, reaching almost 0.18 within 24 hours. This pronounced anisotropy enhancement is ascribed to incorporation of oligomeric hIAPP species with the lipid bilayers consequently inducing greater bilayer rigidity.^46^

Importantly, Fig. 4B,ii reveals that when the DPH/DOPC vesicles were incubated with hIAPP and Tyr-C-dots together, the increase in DPH fluorescence anisotropy was much more subdued – reaching less than 0.12 after 24 hours. This result indicates that the Tyr-C-dots blocked membrane interactions of hIAPP, consistent with the dramatic inhibition of hIAPP-induced cell toxicity (*e.g.*Fig. 4A). In contrast, the bar diagram in Fig. 4B,iii shows that Phe-C-dots did not inhibit hIAPP/bilayer interactions since the recorded DPH anisotropy was similar to the control vesicles incubated with hIAPP alone (*e.g.*Fig. 4B,i). This observation echoes the cell toxicity results indicating that Phe-C-dots did not block hIAPP-associated toxicity (Fig. 4A). It should be noted that the C-dots alone did not have a noticeable effect upon DPH anisotropy (black bars in Fig. 4B).

**Fig. 5:**
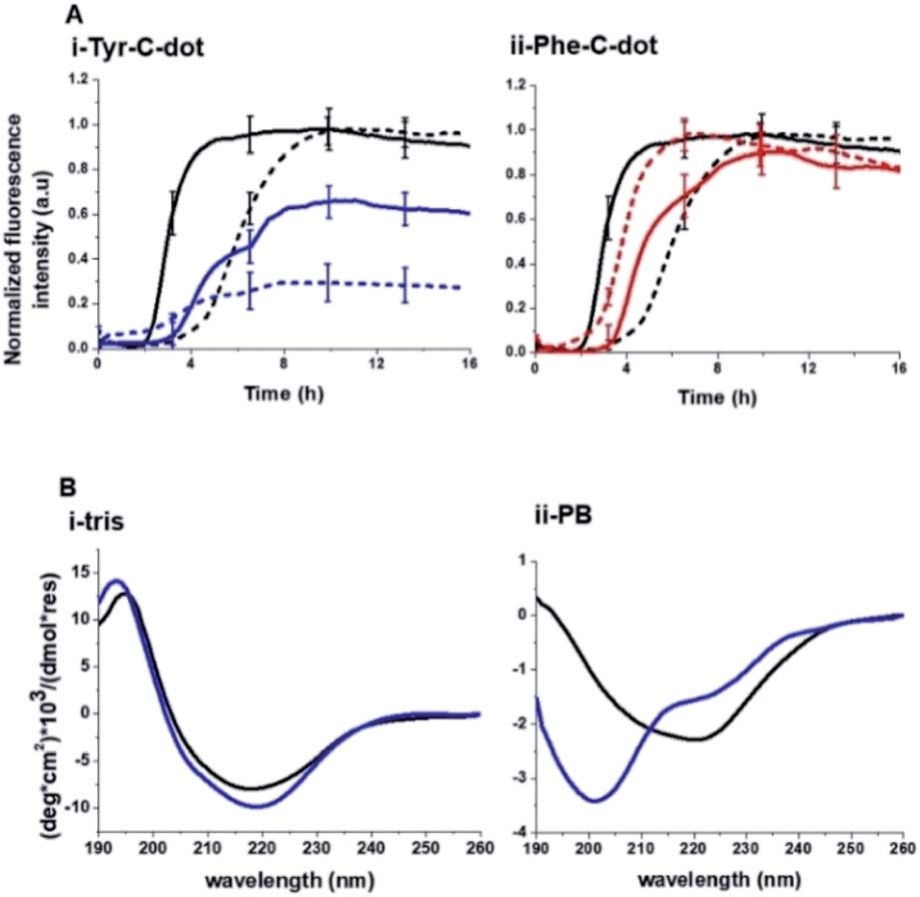
Contribution of hydrogen bonding to carbon dot modulation of hIAPP fibrillation. (A) ThT fluorescence curves recorded in Tris buffer (solid curves) and PB buffer (broken curves). hIAPP alone (black curves); hIAPP + Tyr-C-dots (concentration of 0.03 mg mL^−1^; blue curves); hIAPP + Phe-C-dots (concentration of 0.03 mg mL^−1^; red curves). (B) CD spectra of hIAPP alone (black spectra) and together with Tyr-C-dots (concentration of 0.05 mg mL^−1^; blue spectra) recorded in Tris buffer or PB buffer. Each spectrum was acquired after 24 hour incubation of the peptide or peptide/C-dot mixture.

**Fig. 6:**
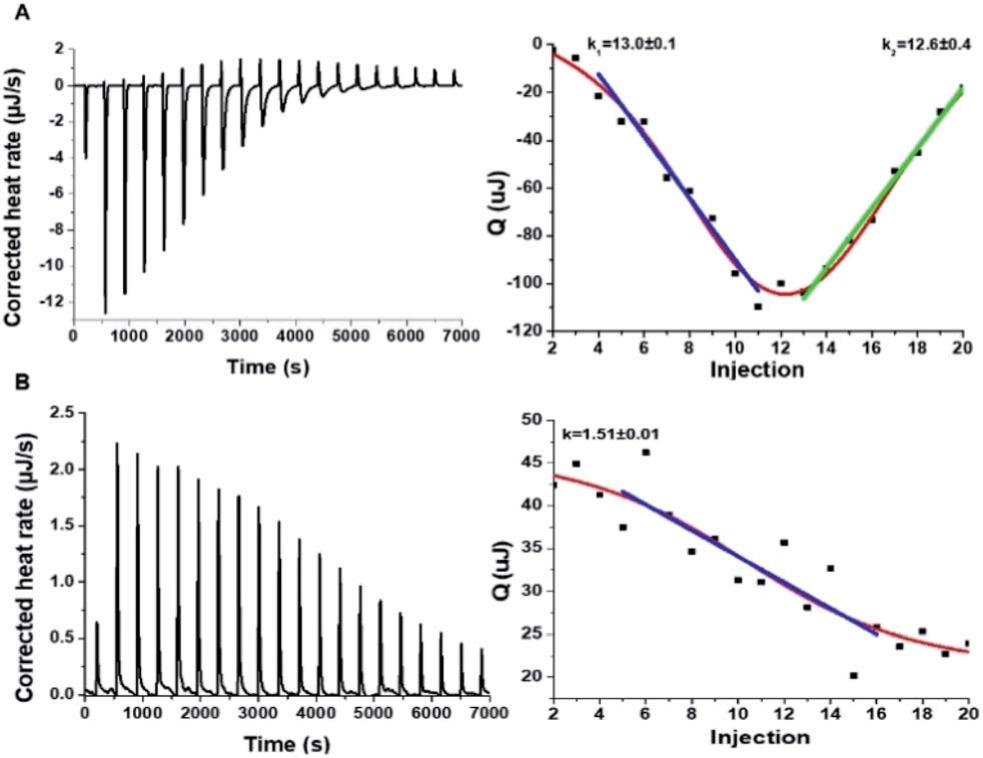
Interactions between hIAPP and the carbon dots in Tris buffer. Isothermal titration calorimetry (ITC) data recorded for: (A) Tyr-C-dot/hIAPP; (B) Phe-C-dot/hIAPP. Left: C-dot/hIAPP titration (after subtraction of the buffer). Right: Heat capacity *vs.* injection numbers.

To elucidate the mechanistic aspects of Tyr-C-dot interference in hIAPP fibrillation, lipid interactions and biological activity, particularly the functional role of the phenolic residues, we analyzed the effects of the C-dots upon hIAPP in Tris buffer (Fig. 5 and 6), rather than the PB buffer employed in the experiments summarized in Fig. 2 and 3.

Notably, Tris buffer largely inhibits hydrogen bonding among solute molecules since solubilized molecules are instead hydrogen-bonded with the OH and NH residues of the Tris molecules.^51,52^ Indeed, the ThT fluorescence curves in Fig. 5A reveal a significant difference between the effects of Tyr-C-dots and Phe-C-dots upon hIAPP fibrillation in Tris buffer *vs.* PB buffer. Fig. 5A demonstrates that fibrillation of hIAPP alone occurred faster (*i.e.* shorter lag time) in Tris buffer than in PB buffer since the absence of ions in Tris buffer promotes interactions between the monomers and association into fibrillar structures.^53,54^

Notably, lesser inhibition of hIAPP fibril formation by Tyr-C-dots was apparent in Tris buffer compared to PB buffer (reflected in the higher ThT fluorescence intensity in Tris buffer than in PB buffer, Fig. 5A,i). This result attests to the prominent role of the phenolic OH residues in inhibiting fibrillation, as the Tris molecules likely block the inhibition effect through hydrogen bonding with the phenol moieties. TEM analysis confirmed that Tyr-C-dots had no effect on hIAPP fibril morphology when incubated in Tris buffer (Fig. 5, SI†). In the case of Phe-C-dots, the effects upon hIAPP fibrillation were small and were hardly impacted by the choice of buffer (Fig. 5A,ii) consistent with the absence of hydrogen bonding involving the phenyl residues of the Phe-C-dots. The CD data in Fig. 5B further confirm that inhibition of hIAPP fibril formation did not occur when Tyr-C-dots were present in Tris buffer, as the β sheet CD trace (exhibiting the signature minimum around 215 nm) was not disrupted by the Tyr-C-dots (Fig. 5B,i). In PB buffer, however, Tyr-C-dots clearly inhibited β-sheet formation (Fig. 5B,ii). Together, the ThT and CD analyses in Fig. 5 underscore the significant contribution of the phenolic residues to the interference of Tyr-C-dots in hIAPP fibril assembly.

The ITC results obtained for the C-dot/hIAPP mixtures in Tris buffer (Fig. 6) furnish additional evidence for the significance of hydrogen bonding involving the phenolic OH residues in the Tyr-C-dot fibril inhibitory effects. Notably, while the heat capacity *vs.* injection numbers curve for Tyr-C-dots in Fig. 6A shows a V shape similar to the ITC results obtained in Tris buffer (*e.g.*Fig. 4A), the endothermic process occurred almost instantly in the titration, different than the initial plateau recorded in the Tyr-C-dot/hIAPP titration in PB buffer (Fig. 4A). This difference is ascribed to the formation of ubiquitous hydrogen bonds between the C-dots and Tris solvent molecules, concomitantly reducing the concentration of sites available for initial hIAPP-C-dot interactions.

Interestingly, the slope corresponding to the initial endothermic binding process in Tris buffer – 12.6 – (Fig. 6A) is significantly smaller than the corresponding slope calculated in PB (*k*_1_ = 27, Fig. 4A). This result is similarly consistent with formation of hydrogen bonding between the Tyr-C-dots and Tris, thereby attenuating the affinity of the C-dots to hIAPP. Indeed, the occurrence of hydrogen bonding between Tris molecules and the C-dots also likely explains the smaller binding constant calculated in the ITC experiments for Phe-C-dots/hIAPP in Tris buffer, 1.5 (Fig. 6B) compared with 19.2 (Fig. 4B), since also the Phe-C-dots exhibit OH and COOH residues on their surface.

## Conclusions

This work explores the inhibitory activity of C-dots synthesized from citric acid and tyrosine against fibrillation of hIAPP. The thrust of this study is the “C-dot structural memory” concept, manifested here in the display of ubiquitous phenolic residues on the C-dots' surface. Spectroscopic, microscopic and thermodynamic experiments as well as cell viability analysis demonstrate that the Tyr-C-dots effectively mimicked the fibril inhibition properties of natural polyphenols. Interestingly, the experiments indicate that C-dots prepared from tyrosine exhibited significant fibril disruption activity compared to C-dots synthesized from phenylalanine. Indeed, mechanistic analysis reveals that the difference between the C-dots displaying phenol residues and C-dots comprising phenyls can be traced to hydrogen bonding of the hydroxide units in the phenol residues. In conclusion, this work points to a potential use of Tyr-C-dots as a therapeutic platform in type-II diabetes and other protein misfolding diseases.

## Conflicts of interest

There are no conflicts to declare.

## Supplementary Material

NA-002-D0NA00870B-s001
