# Supplementary material for: Tyrosine carbon dots inhibit fibrillation and toxicity of the human islet amyloid polypeptide
Source: Nanoscale Adv. 2020 Nov 10;2(12):5866–73. doi: 10.1039/d0na00870b (PMC9419576; doi:10.1039/d0na00870b)
Supplement: NA-002-D0NA00870B-s001 [file NA-002-D0NA00870B-s001.pdf]

Electronic Supplementary Material (ESI) for Nanoscale Advances.
This journal is © The Royal Society of Chemistry 2020

Tyrosine carbon dots inhibit fibrillation and toxicity of the human Islet amyloid polypeptide

Daniel N. Bloch[1], *Shani Ben Zichri*[1], *Sofiya Kolusheva*[2], *Raz Jelinek*[1,2]

[1]Department of Chemistry and [2] Ilse Katz Institute for Nano-Science and Technology (IKI),

Ben Gurion University of the Negev, Beer Sheva 84105, Israel

Supporting Information

Experimental

X-ray photoelectron spectroscopy (XPS)

Concentrated solutions of C-dots were dropcasted on silicon wafers and measurements were performed using an X-ray photoelectron spectrometer ESCALAB 250 ultrahigh vacuum ($1 * 10^{-9}$ bar) apparatus with an AlKa X-ray source and a monochromator. The beam diameter of was 500 mm and with pass energy (PE) of 150 eV survey spectra were recorded, while for high energy resolution spectra the recorded pass energy (PE) was 20 eV. The AVANTGE program was used to process the XPS results.

Fibril disintegration

hIAPP sample at a concentration of 35uM in 10 mM phosphate buffer 0.05M (pH 7.4) was incubated at 25 ° C for 24 h while shaking to create fibrils, after the incubation ThT was added for example at a final concentration of 10uM and the sample was divided into two, Tyr-C-dot where added to half from the example In the final concentration of 0.5mg/mL and to the second half buffer was added in the relevant volume/ The fluorescence intensity was measured immediately after the addition of Tyr-C-dot and after an additional 24 hours of incubation. ThT fluorescence measurements were taken at 25 °C using 96-well black plate on a Biotek Synergy H1 plate reader at λex = 440 and λem = 485 nm. The self-fluorescence of the C-dots was subtracted from measured fluorescence for each sample. Results are presented as means ± SD for each sample. From these samples grids for TEM were prepared at the end of the experiment

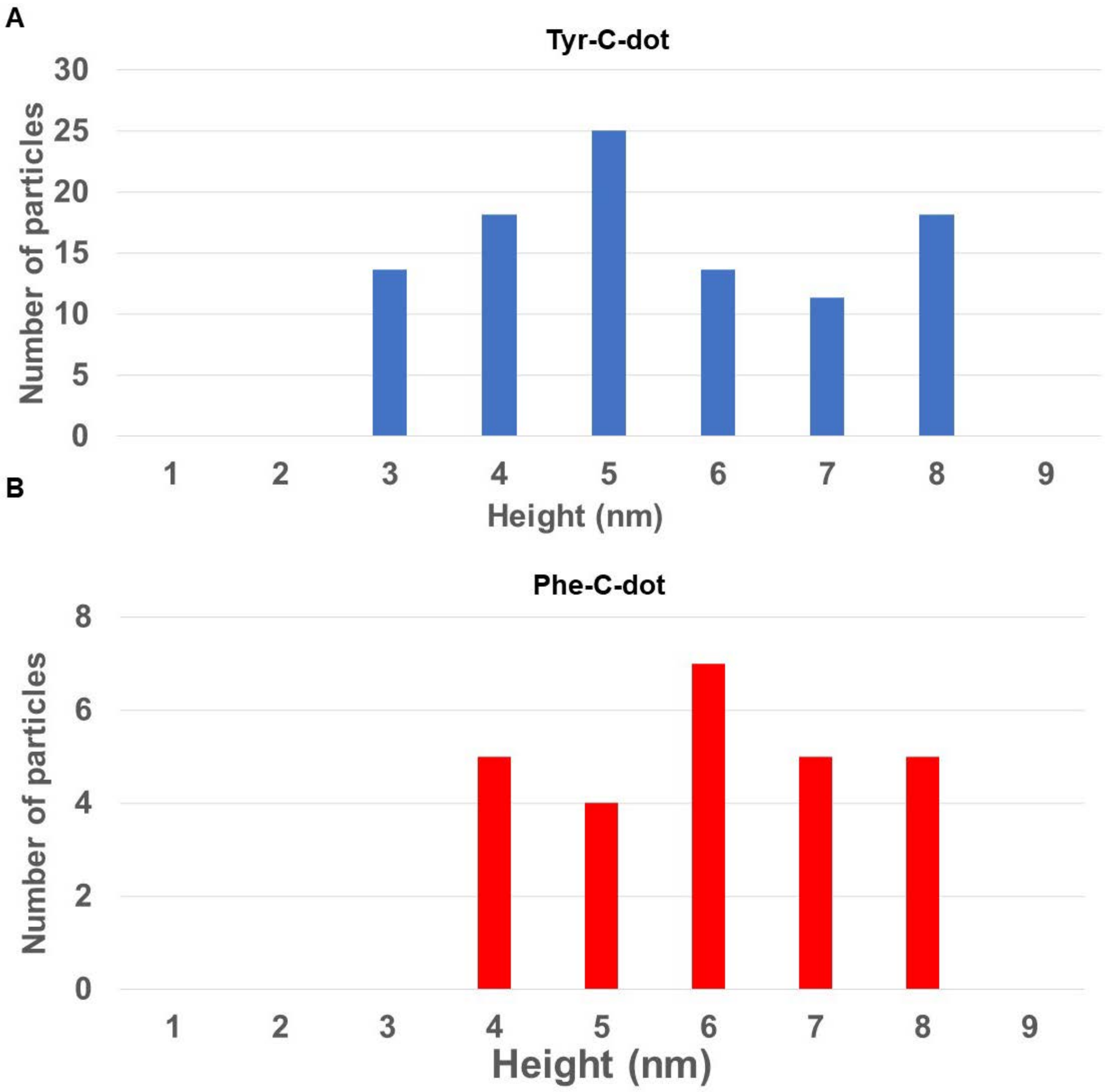

Figure 1,SI: Histograms showing the height distribution of Tyr C-dot (A) and Phe C-dot (B) as measured by AFM measurements. For the Tyr-C-dot the average height is approximately 4.9nm (n=44) and for the Phe-C-dot average height is approximately 5.7nm (n=26)

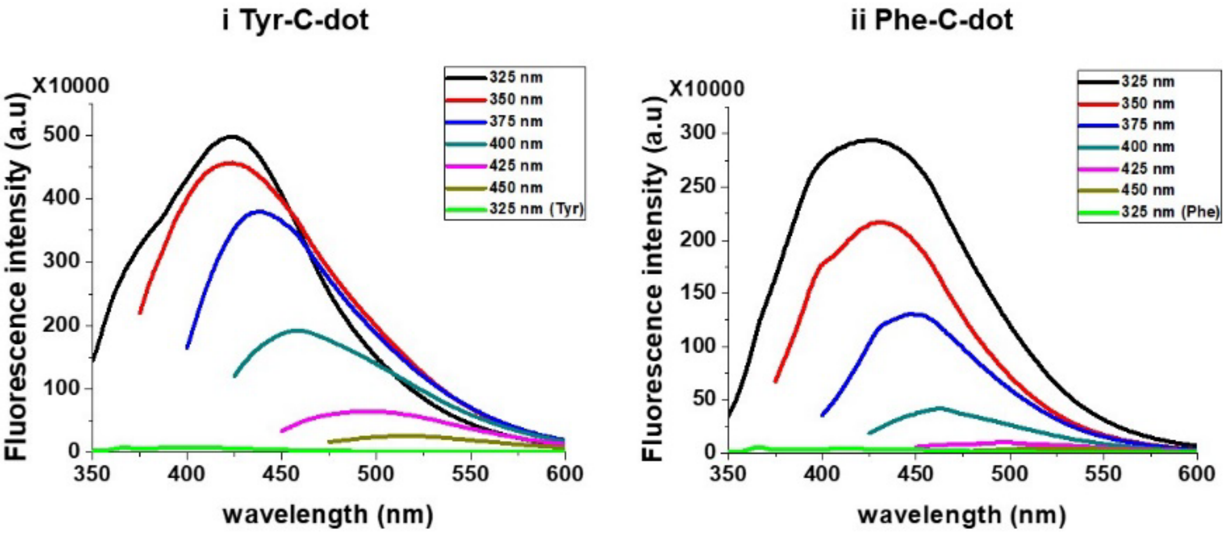

Figure 2,SI: Fluorescence emission spectra of Tyr-C-dot and Phe-C-dot showing excitation dependent emission property of the C-dots. the green curve shows the emission of the precursor in the same excitation who demonstrated the maximum emission of the C-dot.

A Tyr-C-dot

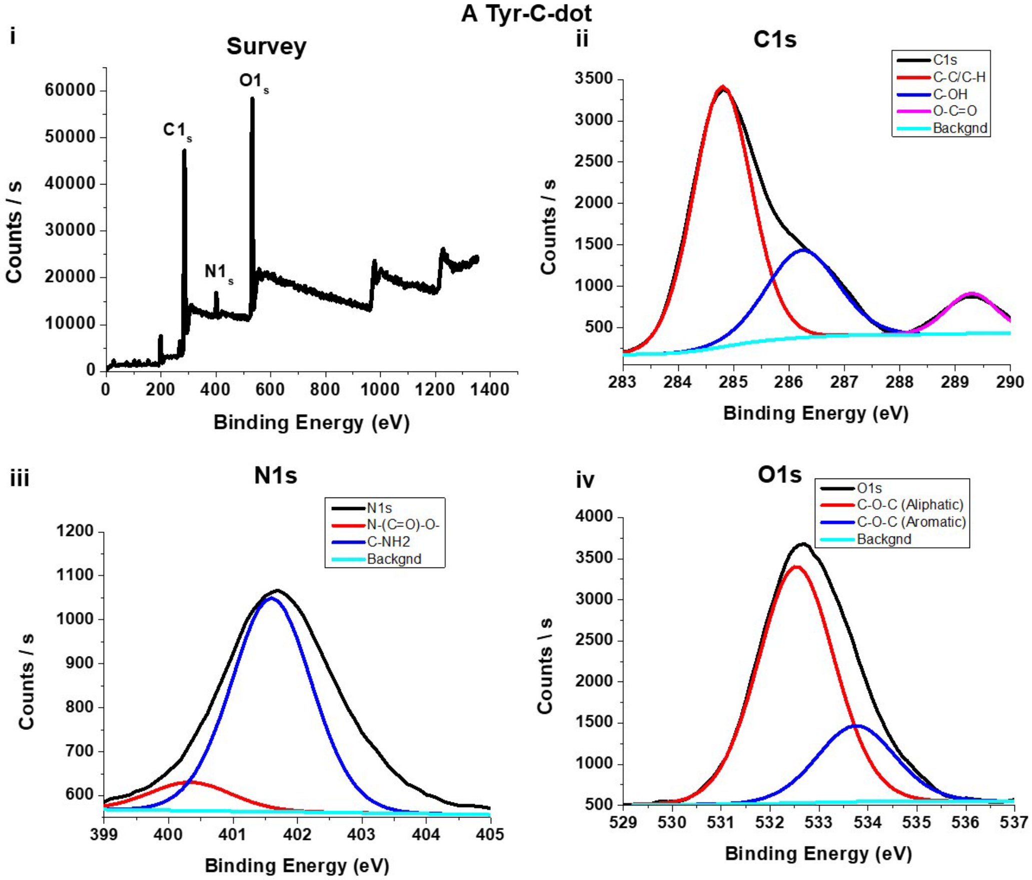

B Phe-C-dot

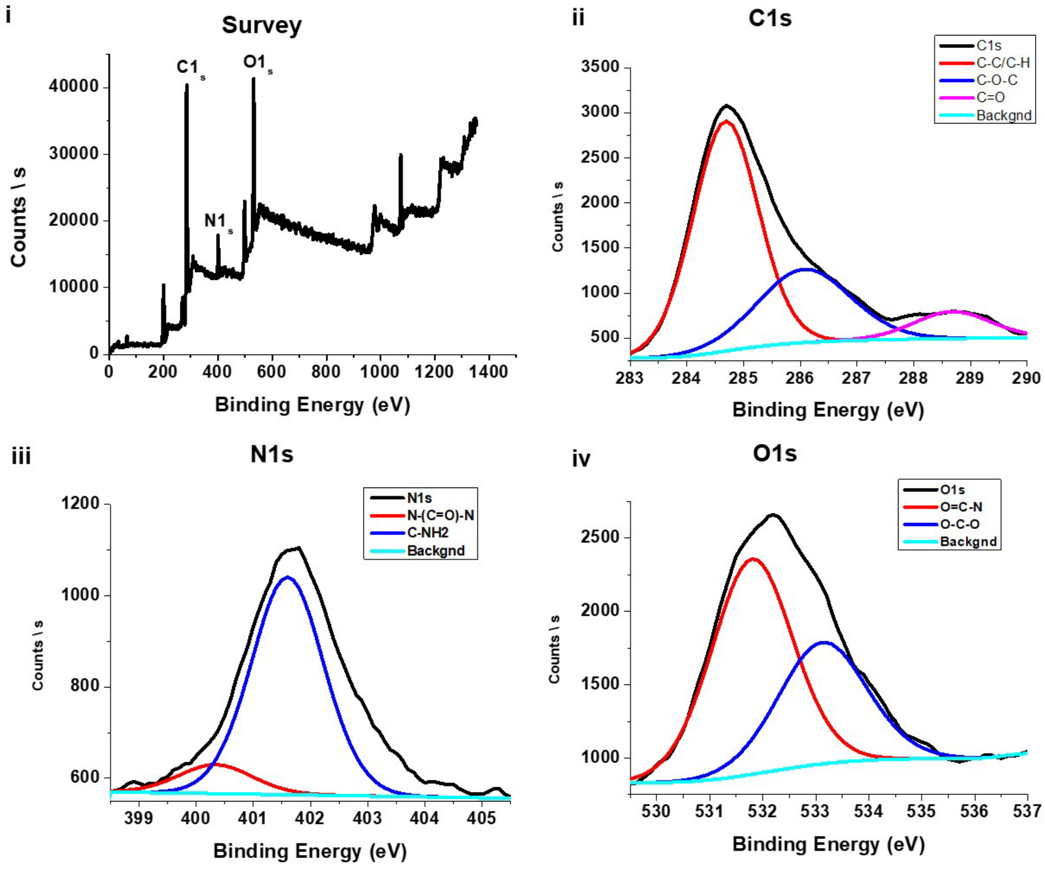

Figure 3,SI: X-ray photoelectron spectroscopy (XPS) analysis: A. Tyr-C-dots. i. XPS full scan survey of Tyr-C-dots indicates peaks for C1s, N1s and O1s at 285, 401 and 532 eV respectively. ii. C1s spectrum shows three peaks, at 284.7 eV for C-C/C=C, 286.2 eV for C-OH and 289.3 eV for O-C=O. iii N1s spectrum showing two peaks at 400.3 and 401.67 eV corresponding to pyridinic, N-(C=O)-O- and C-NH2 respectively and iv O1s spectrum of C-dots showing two peaks at 532.5 and 533.8 indicates presence of aliphatic and aromatic C-O-C respectively.

B. Phe-C-dots. i. XPS full scan survey of Phe-C-dots indicates peaks for C1s, N1s and O1s at 285, 401 and 532 eV respectively. ii. C1s spectrum shows three peaks, at 284.7 eV for C-C/C-H, 286.1 eV for C-O-C and 288.7 eV for C=O. iii. N1s spectrum showing two peaks at 400.5 and 401.6 eV corresponding to pyridinic, N-(C=O)-N- and C-NH2 respectively and iv. O1s spectrum of C-dots showing two peaks at 531.8 and 533.2 indicates presence of C=O-N and O-C-O respectively.

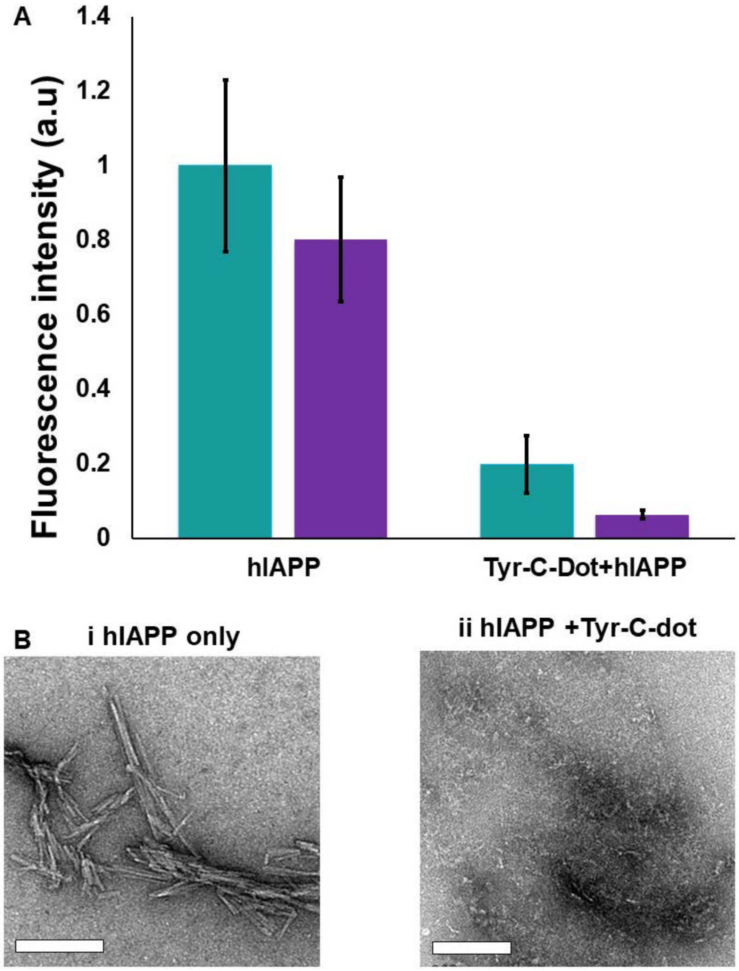

Figure 4,SI: A bar graph showing the normal fluorescence of ThT obtained as a result of being subtracted from the hIAPP fibrils before and after the addition of Tyr-C-dot, the Tyr-C-dot was added to the protein after being incubated alone for 24 h in order to form fibrils. The turquoise column is a measurement taken immediately after the addition of the Tyr-C-dot and the purple is a measurement made after 24h of incubation between the fibrillation of the protein and the c-dot, as can be seen already with the addition of the C-dot there is a decrease in fluorescence, i.e. there is destruction of the fibrils, this trend Also visible after 24h.

B. TEM images taken after the 24h incubation, in the control measurements an accumulation of long fibrils can be seen, however an image of the protein after the addition of Tyr-C-dot after 24h of an incubation together with the fibrillated protein no fibrillation can be seen at all, although small aggregates can be seen. Bras correspond to 200nm.

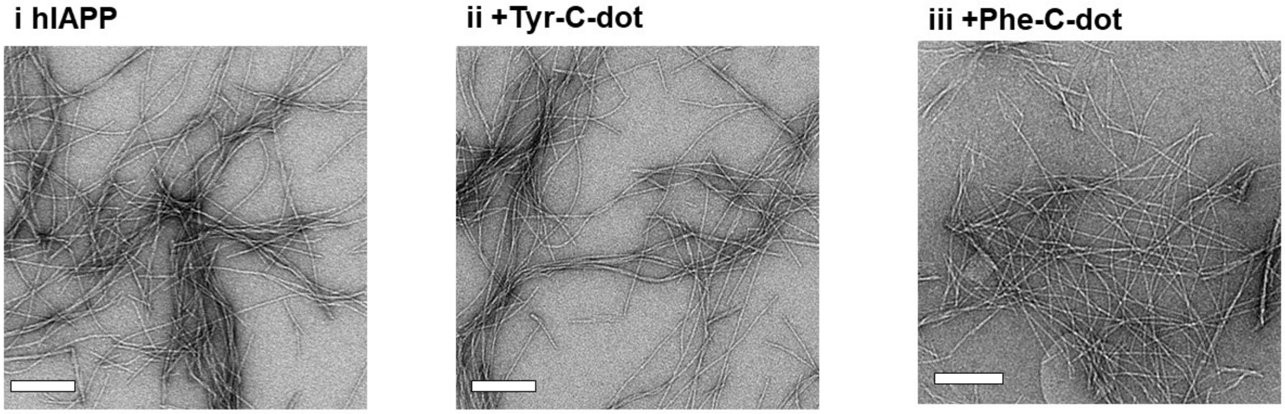

Figure 5,SI: TEM images of i-hIAPP, ii- hIAPP+Tyr-C-dot and iii-hIAPP+Phe-C-dot in tris. Bars correspond to 200nm
